# The Eldgjá eruption: timing, long-range impacts and influence on the Christianisation of Iceland

**DOI:** 10.1007/s10584-018-2171-9

**Published:** 2018-03-19

**Authors:** Clive Oppenheimer, Andy Orchard, Markus Stoffel, Timothy P. Newfield, Sébastien Guillet, Christophe Corona, Michael Sigl, Nicola Di Cosmo, Ulf Büntgen

**Affiliations:** 1 Department of Geography, University of Cambridge, Cambridge, UK; 2 Faculty of English, University of Oxford, Oxford, UK; 3 Institute for Environmental Sciences, University of Geneva, Geneva, Switzerland; 4 Dendrolab.ch, Department of Earth Sciences, University of Geneva, Geneva, Switzerland; 5 Departments of History and Biology, Georgetown University, Washington, DC USA; 6 Geolab UMR6042 CNRS, Université Blaise Pascal, Clermont-Ferrand, France; 7 Laboratory of Environmental Chemistry, Paul Scherrer Institute, 5232 Villigen, Switzerland; 8 Institute for Advanced Study, Princeton, NJ USA; 9 Princeton University, Princeton, NJ USA; 10 Swiss Federal Research Institute WSL, Birmensdorf, Switzerland; 11 Global Change Research Centre and Masaryk University, Brno, Czech Republic

## Abstract

The Eldgjá lava flood is considered Iceland’s largest volcanic eruption of the Common Era. While it is well established that it occurred after the Settlement of Iceland (circa 874 CE), the date of this great event has remained uncertain. This has hampered investigation of the eruption’s impacts, if any, on climate and society. Here, we use high-temporal resolution glaciochemical records from Greenland to show that the eruption began in spring 939 CE and continued, at least episodically, until at least autumn 940 CE. Contemporary chronicles identify the spread of a remarkable haze in 939 CE, and tree ring-based reconstructions reveal pronounced northern hemisphere summer cooling in 940 CE, consistent with the eruption’s high yield of sulphur to the atmosphere. Consecutive severe winters and privations may also be associated with climatic effects of the volcanic aerosol veil. Iceland’s formal conversion to Christianity dates to 999/1000 CE, within two generations or so of the Eldgjá eruption. The end of the pagan pantheon is foretold in Iceland’s renowned medieval poem, *Vǫluspá* (‘the prophecy of the seeress’). Several lines of the poem describe dramatic eruptive activity and attendant meteorological effects in an allusion to the fiery terminus of the pagan gods. We suggest that they draw on first-hand experiences of the Eldgjá eruption and that this retrospection of harrowing volcanic events in the poem was intentional, with the purpose of stimulating Iceland’s Christianisation over the latter half of the tenth century.

**Electronic supplementary material:**

The online version of this article (10.1007/s10584-018-2171-9) contains supplementary material, which is available to authorized users.

## Introduction

**Fig. 1:**
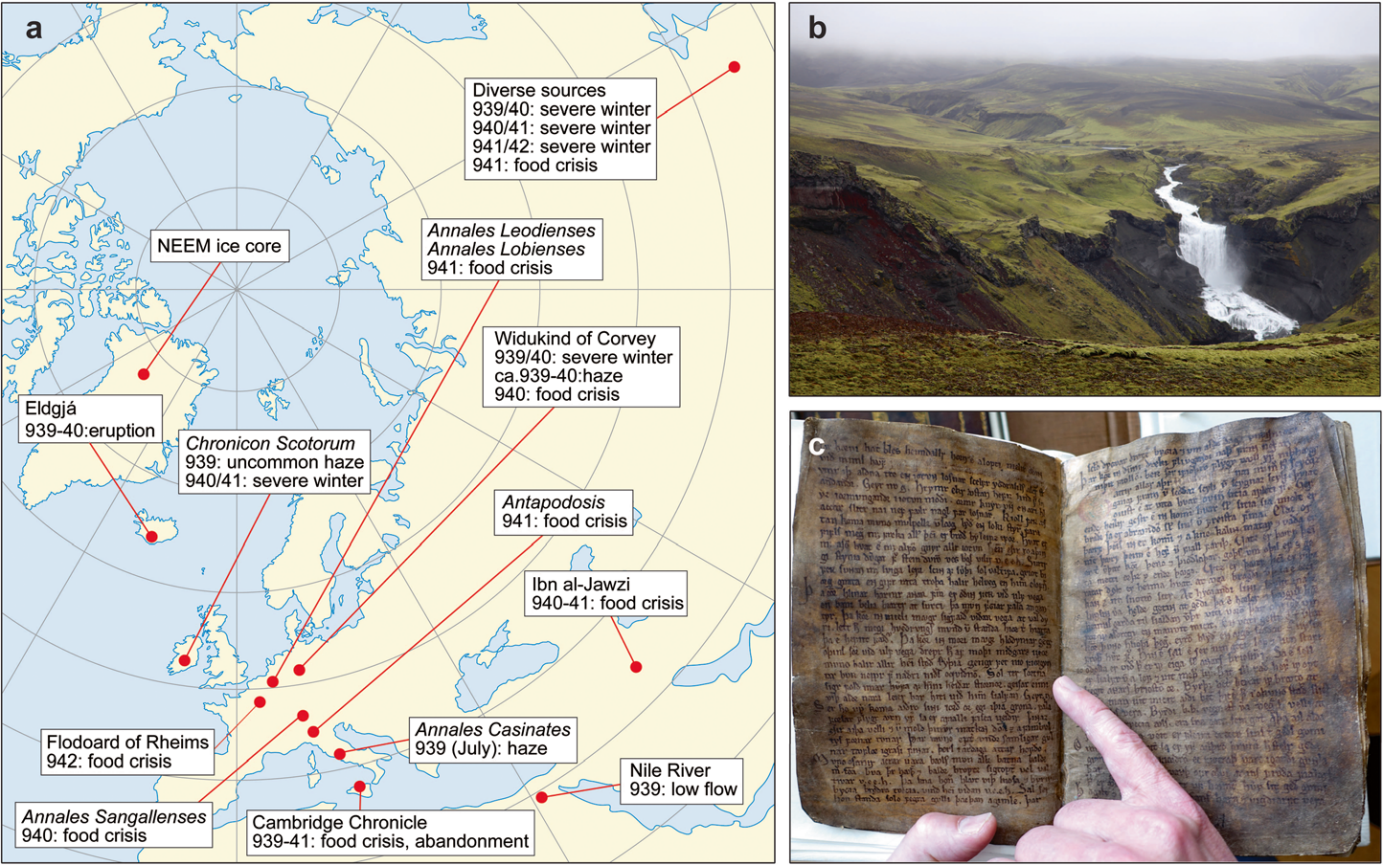
**a** Locations of Eldgjá, the NEEM drill site and medieval documentary evidence referenced in the text covering 939–942 CE. **b** Section of the 75-km-long Eldgjá eruption fissure at Ófærufoss. **c** The Codex Regius; finger points to beginning of stanza 57 of *Vǫluspá*

The Eldgjá (‘fire gorge’) eruption occurred along an approximately 75-km-long fissure system associated with Katla volcano, which lies beneath the Mýrdalsjökull ice cap (Fig. 1). It is the largest known lava flood eruption of the Common Era—an estimated 19.6 km^3^ of magma (dense-rock equivalent) were erupted, mostly effusively, accompanied by the emission of 30–70 Tg of SO_2_ into the atmosphere (Thordarson et al. 2001). These figures compare with the 14.7 km^3^ of lava and tephra output (dense-rock equivalent; Thordarson and Self 1993) and 122 Tg of SO_2_ emission (Thordarson et al. 1996) reckoned for the 1783–1784 Laki eruption. Despite its size and its known occurrence after the ‘Settlement’ of Iceland in circa 874 CE (Eldgjá’s deposits lie stratigraphically above the so-called Settlement tephra layer of the 870s CE; Grönvold et al. 1995; Thordarson et al. 2001; Schmid et al. 2016), the date of the Eldgjá eruption has remained unclear (Larsen 2000; Óladóttir et al. 2008; Baillie and McAneney 2015). No direct references to it have hitherto been noted in contemporary sources. The *Landnámabók* (Iceland’s ‘Book of Settlements’; Benediktsson 1968), first written in the twelfth century, appears to document the aftermath of the Eldgjá eruption but does not provide a clear date. It records that the first settlers in the area of the eruption site were Ásbjǫrn Reyrketilsson and his brother Steinfiðr. Ásbjǫrn is noted as a particular devotee of the pagan god Thor, to whom he dedicated his part of the settled land, by designating it Þórsmǫrk (‘Thor’s wood’). This area seems later to have been abandoned, likely as a result of the eruption. It is explicitly described as a ‘wasteland’ in other twelfth-century sources (the Icelandic word is *eyði* and is referred to by Benediktsson 1968).

Published estimates for the eruption date are 934 ± 2 CE (Hammer et al. 1980), 938 ± 4 CE (Zielinski et al. 1995) and 933 ± 1 CE (Vinther et al. 2006). They are based on analyses of Greenland ice cores which preserve both sulphate and tephra fallout associated with the eruption. The most widely quoted date in the literature is 934 CE and draws on evidence from ice cores and medieval sources (Stothers 1998). Another date that has been proposed is 939 CE based on observation of a blood-red Sun as recorded in an Irish chronicle, suggestive of a manifestation of volcanic haze (McCarthy and Breen 1997). This attribution to Eldgjá was assumed correct by Sigl et al. (2015) and used as tie point in their revision of the Greenland Ice Core Chronology 2005 (GICC05) timescale in this period. With this evident uncertainty in the literature on Eldgjá, it remains critical to confirm when the eruption occurred.

We revisit the questions of the timing and consequences of the Eldgjá eruption by exploiting a serendipitously-fixed chronological tie-point for the NEEM-2011-S1 ice core from northern Greenland and the high-resolution glaciochemical record that the core provides. The marker concerned is the ‘Millennium Eruption’ of Changbaishan volcano (also called Tianchi, Baitoushan and Paektu, and located on the border between China and DPR Korea), now known to have occurred in late 946 CE (Oppenheimer et al. 2017). The fallout from this event (including tephra) is clearly identified in the aforementioned ice core (Sun et al. 2014), as is that from the Eldgjá eruption, enabling us to make a precise calculation of the timing of the latter event. With the secure date, we investigate the eruption’s climatic impacts and associated environmental and societal consequences. We conclude by drawing attention to one of Iceland’s most celebrated medieval poems, *Vǫluspá*, which appears to record the Eldgjá eruption.

## Methods

The foundation of our dating analysis is a high-resolution dataset for the NEEM-2011-S1 ice core from northern Greenland drilled at 77.45° N, 51.06° W (Jensen et al. 2014), approximately 1900 km northwest from Katla volcano. We have focused on a section of the core that contains the signatures of both the Eldgjá eruption and the Millennium Eruption of Changbaishan. In addition to non-sea-salt sulphur (nssS), calcium (Ca) and chlorine (Cl) concentrations, determined by inductively coupled plasma mass spectrometry (Sigl et al. 2013), we carried out a replicate analysis on a parallel ice-core section (Oppenheimer et al. 2017). NEEM-2011-S1 data coverage (by depth) for the section of interest in the mid-tenth century was 91% for the original continuous flow analysis (Sigl et al. 2013; Jensen et al. 2014) but we achieved 96% with our replicate analysis, improving definition of seasonal cycles. The largest remaining data gap is 10 cm (corresponding to less than 6 months—annual layers are approximately 20.5 cm thick) and situated 3 years before the Millennium Eruption signature. This arose from removal of a fracture that could have been contaminated with core drilling fluid.

We also generated reconstructions of Northern Hemisphere summer temperature anomalies (NH1, NH2) using a dataset of 14 tree-ring widths and 16 maximum-density clusters (Stoffel et al. 2015). The latter contain less biological memory, compared to tree-ring width data, and are thus better suited to assess the interannual climate variability associated with volcanic forcing. NH1 is based on a bootstrap linear model using principal component analysis to calibrate 30 tree-ring regional clusters against a mean of observational June–July–August (JJA) 40°–90° N temperature (1805–1976) over land from the Berkeley Earth Surface dataset (BEST). To account for decreasing chronologies back in time, a nested approach was applied. The final reconstruction was achieved by splicing all the nested time series (*n* = 32), and after the mean and variance of each nested reconstruction segment had been adjusted to the best replicated nest (1775–1976). Calibration and validation statistics (*R*^2^ = 0.25–0.51, *r*^2^ = 0.19–0.45, reduction of error = 0.20–0.47, coefficient of efficiency (ce) = 0.18–0.45) confirm the predictive skill of the reconstruction.

NH2 is an alternative reconstruction based on a two-step procedure. First, linear regression analysis was applied to calibrate the cluster series to JJA-gridded temperature anomalies (with respect to 1961–1990) from the Climatic Research Unit (CRU, University of East Anglia) and BEST datasets. For each cluster, we kept either the CRU or BEST reconstruction, depending on the significance level of the verification statistics (reduction of error, coefficient of efficiency). Seven reconstructions failing classical calibration and verification tests were excluded from the final NH2 reconstruction. Averaging was performed to composite 23 cluster reconstructions into the NH2 chronology.

To enhance the signal-to-noise ratio and to quantify the reconstructed cooling in the context of climate variability prevailing at the time of the eruptions investigated here, we transformed JJA values of the NH1 and NH2 records into temperature anomalies. This was achieved with a 31-year moving average time window as a reference to compute the yearly anomalies (for 924–954 CE, 931–961 CE and 1768–1798, so as to span both the Eldgjá and Laki eruptions).

In order to map the spatial extent of the cooling induced by the Eldgjà and Laki eruptions, we used a gridded reconstruction developed by Guillet et al. (2017). (The Laki eruption was investigated for comparative purposes, given its similarities with the Eldgjá eruption.) Only grid points with a coefficient of efficiency exceeding 0.1 were used for the spatial representation of temperature anomalies.

## Results and discussion

### Timing and duration of the eruption

**Fig. 2:**
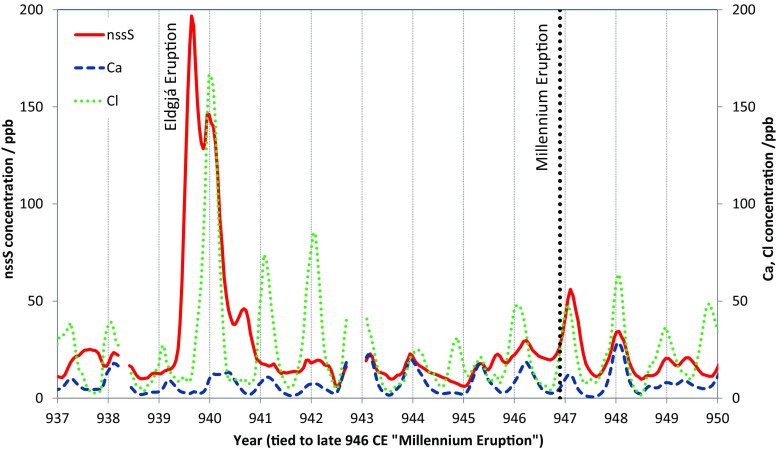
Chlorine (Cl), calcium (Ca), and non-sea salt sulphur (nssS) concentrations in the NEEM-2011-S1 ice core. The timescale is pinned to the late 946 CE date for the Millennium Eruption (Oppenheimer et al. 2017) and is equivalent to the NS1-2011 timescale (Sigl et al. 2015). The time lag between initial increases in nssS linked to Eldgjá and the Millennium Eruption is 7.5 years. The modest nssS spike attributed to the Millennium Eruption is co-located with glass shards that have been chemically matched to type materials from the Millennium Eruption (Sun et al. 2014)

Figure 2 shows the sub-annual resolution chlorine (Cl), calcium (Ca) and non-sea salt sulphur (nssS) stratigraphy for the NEEM-2011-S1 core. The Millennium Eruption sulphur anomaly, confirmed by the presence of identifiable ash (Sun et al. 2014), is modest. Far more striking is a pronounced nssS signal that begins 7.5 years earlier, i.e. in 939 CE (Fig. 2). This can be firmly attributed to Eldgjá because the prominent horizon is also present in the GISP2 ice core, from which glass shards have been extracted and matched geochemically to Eldgjá compositions (Zielinski et al. 1995).

Seasonality is pronounced for Ca (typically peaking in late winter/spring; Kang et al. 2015) and Cl (peaking in midwinter and associated with long-range transport of marine aerosol from the sea surface and/or from sea ice by North Atlantic storms; Davidson et al. 1989; Rhodes et al. 2017). A smaller summer peak in Cl occurs around July (Davidson et al. 1989), attributed to sea spray from Greenland coastal areas. These seasonal signals pinpoint the timing and prolongation of the Eldgjá eruption. The volcanic sulphur signal picks up strongly as the 938/939 CE winter Cl and Ca peaks wane but before the summer 939 CE Cl peak. We cannot be certain that the initial deposition indicated in the record derives from the first phase of the eruption, but if it does, it points to an eruption onset in spring 939 CE, assuming transport of aerosol between source and sink of a few weeks at most. Sulphur concentration reaches a peak before the 939/940 CE winter Cl spike, then falls and then rises again coincident with the winter 939/940 CE Cl peak. Sulphur abundance declines in early 940 CE but remains elevated through much of the year. There is a subsidiary sulphur spike before the winter 940/941 CE Cl peak, suggestive of the eruption’s persistence to around autumn 940 CE. While it is possible that the eruption continued after this time without further deposition to northern Greenland, it appears to have been significantly shorter (with episodic activity spanning around 1.5 years) than has previously been suggested. For instance, Zielinski et al. (1995) considered a 3–6-year duration based on sulphate measurements for the GISP2 ice core, while Thordarson et al. (2001) entertained episodic activity spanning up to 8 years. This matter is pertinent to the timeline and intensity of sulphur emission to the atmosphere and thus for understanding atmospheric and climatic change resulting from the eruption.

One striking feature of the glaciochemical signal is the high magnitude of the Cl spike in the 939/940 CE winter. Inspection of the Na record (not shown here) indicates that the Cl abundance exceeds that which can be accounted for by sea salt deposition, pointing to a contribution from volcanic fallout. This is confirmed by the presence of trace metals such as thallium and bismuth (not shown here). This winter Cl spike is accompanied by a peak in S whereas the initial S deposition (spring-to-autumn 939 CE) is not coupled with a Cl anomaly. Zielinski et al. (1995) noted the same sequence in their analysis of the GIPS2 ice core (recovered at a site in central Greenland, more than 600 km from the NEEM drill site): sulphate preceding chloride deposition. The tephra they extracted from the GISP2 core were co-located within the high chloride section.

These observations and comparisons are puzzling. The variable combinations of sulphur, chlorine and tephra in the Greenland ice could reflect source conditions and/or plume chemistry and transport. The Eldgjá products are dominated by lava flows but include an estimated 1.3 km^3^ (dense-rock equivalent) of tephra associated with at least 16 explosive phases of the eruption, sourced at various points along the fissure system (Moreland 2017). These explosive phases included sub-glacial phreatomagmatic eruptions earlier in the eruption history, which gave way to magmatic eruptions as the fissure system extended well beyond the ice cap (Moreland 2017). This progression may well be reflected in the nature and content of tephra and volatile species carried in the volcanic cloud and in the scavenging of volatiles by hydrometeors and ash particles. However, we also have to consider that we lack information on the chemistry (including photochemistry) and transport of the plume during the course of the eruption. In particular, we do not know the transit times, trajectories or heights of the plumes that deposited materials to the surface at the ice core sites.

The high amplitudes of the winter 940/941 and winter 941/942 Cl peaks might be explained by increased sea ice (a significant source of Na and Cl to the interior of Greenland in winter; Rhodes et al. 2017). If correct, this postulated growth in sea ice might be attributable to climate forcing of the Eldgjá eruption and might represent a pattern common to other boreal volcanic eruptions.

### Climatic variation during/after the eruption

**Fig. 3:**
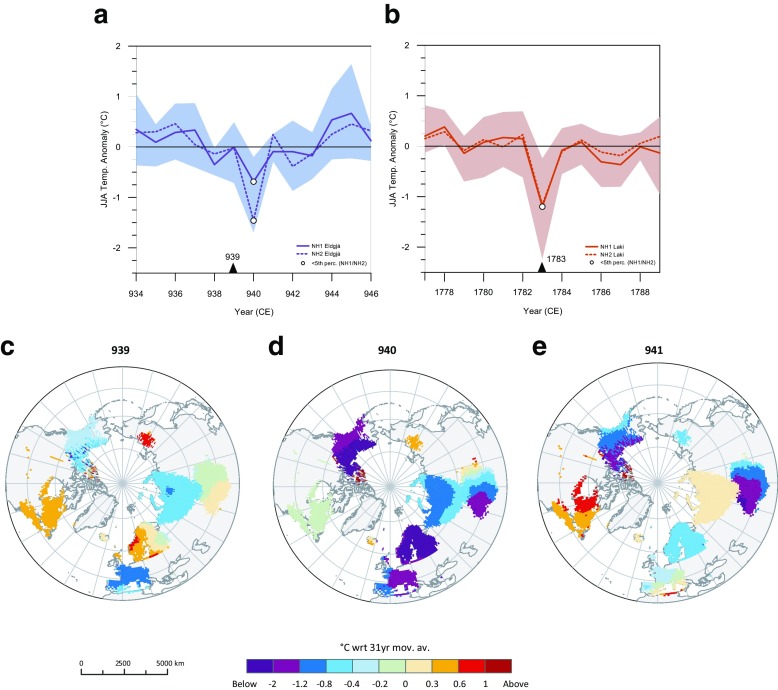
Northern hemisphere summer (JJA) temperature anomalies reconstructed from the two tree-ring chronologies (NH1 and NH2; Stoffel et al. 2015) spanning the eruptions of **a** Eldgjá and **b** Laki. Shaded areas denote uncertainties (2.5 and 97.5 percentiles) related to the NH1 tree-ring reconstruction (Sect. 2). Spatial extent of the JJA temperature anomalies are for **c** 939 CE, **d** 940 CE and **e** 941 CE

We next examine the climate response of the eruption using the two tree-ring-based reconstructions (NH1 and NH2; see Sect. 2; Stoffel et al. 2015). These represent Northern Hemisphere (NH; > 40° N) summer (JJA) land temperatures (Fig. 3a) for the past 1500 years. Cooling associated with Eldgjá amounts to ~ 0.7 °C for NH1 and ~ 1.5 °C for NH2, the 42nd and second largest summer cooling signals, respectively, in the 1500-year-long reconstructions. For comparison, Fig. 3b also shows the reconstructions spanning the period of the 1783–1784 Laki eruption. Summer cooling linked to Laki is similar for both reconstructions, ~ 1.2 °C, the sixth and eighth largest summer cooling in the NH1 and NH2 datasets, respectively. All these anomalies lie below the fifth percentile of coolest years for both reconstructions. JJA-gridded temperature reconstructions are shown for 939, 940 and 941 CE (Fig. 3c–e) and for the years around the 1783–1784 Laki eruption (Fig. 1 in Supplementary Content). In 940 CE, cooling is most pronounced in Central Europe, Scandinavia, the Canadian Rockies, Alaska and Central Asia and reaches − 2 °C (with respect to 1961–1990). Notably, for both Icelandic eruptions, the hemispheric cooling is seen in just one summer (although the anomaly persists in parts of Canada and Central Asia in 941 CE; Fig. 3e). This contrasts with the 2–5-year summer cooling signals associated with large, sulphur-rich explosive eruptions of equatorial volcanoes (Stoffel et al. 2015).

### Annals and chronicles attesting to atmospheric optical phenomena, climatic anomalies and food crises

Our secure date for the eruption focuses attention on an entry in the *Chronicon Scotorum*, an Irish chronicle for 939 CE (McCarthy and Breen 1997): ‘the Sun was of the colour of blood from the beginning of one day to the middle of the following day’ (Supplementary Content). The entry is egregious—nowhere else in the entire chronicle is a blood-red Sun recorded. We find a similar report in the *Annales Casinates*, compiled in an abbey southeast of Rome (Supplementary Content). An entry plausibly dated to mid-July 939 CE reads ‘We looked at the sun, it did not have any strength, neither light nor heat. But we saw the sky and the colour [or ‘appearance’] of it changed, as though viscous. And others said that they saw the sun as though half’ (Supplementary Content). Such descriptions are echoed in observations made eight centuries later as volcanic haze sourced from the 1783–1784 Laki eruption spread across the Mediterranean (Grattan and Brayshay 1995; Thordarson et al. 1996).

The near-contemporary chronicle, *Res Gestae Saxonicae*, written at the abbey of Corvey (east of Paderborn in Germany), similarly records blood-red sunlight and also tells that the winter of 939/940 CE was exceptionally severe (Supplementary Content). Irish annals also attest to a severe winter of 940/941 CE, with rivers and lakes freezing over (Supplementary Content). Chinese sources as well indicate uncommonly severe winters in 939/940 CE, 940/941 CE and 941/942 CE. Remarkably, snow is reported in Inner Mongolia in June 939 CE. Unusual snowfall reoccurred the following winter (939/940 CE) when the Later Jin emperor Shi Jingtang lamented 50 continuous days of snow, which ‘harmed the people’ (Supplementary Content). The next winter was likewise difficult. In early February 941 CE, ice was observed in Qingzhou (modern Shandong province) to extend 100 li (50 km) out from the coast, into the Bohai Sea. The winter of 941/942 CE was as well ‘extremely cold’. In late December 941 CE, the Grand Canal in Jiangdu (modern Jiangsu province) froze over (Supplementary Content). Again, these observations mirror records of the severe winter of 1783/1784 during the Laki eruption (Witham and Oppenheimer 2004), though the relationship with volcanic forcing remains unclear (D’Arrigo et al. 2011; Schmidt et al. 2012; Cole-Dai et al. 2014).

A further point of comparison between these two great Icelandic eruptions is suppressed flow of the Nile—records from the Roda nilometer in Cairo indicate anomalously low discharge in 939 CE (Popper 1951). This phenomenon has been interpreted as a dynamical response of the atmosphere to volcanic aerosol with weakened summer monsoonal precipitation in the source areas of the Nile (Oman et al. 2006; Manning et al. 2017).

These reports of climatic anomalies coincide with food shortages from 940 CE recorded in a variety of narrative sources (Supplementary Content; Newfield 2015), implicating the Eldgjá eruption in substantial reductions in arable output in some regions of Western and Eastern Eurasia for which evidence is extant. For instance, the *Ta’rīkh Jazīrat Şiqilliya* or *Cambridge Chronicle*, a tenth- or eleventh-century list of events in Sicily, recounts a dire subsistence crisis in AH 328 (939–940 CE) that led to desertion and abandonment of fortresses and countryside (Amari 1880). In his *Antapodosis*, written circa 950 CE, Liudprand of Cremona mentions a food shortage that ‘devastated Italy’ in 941 CE (Supplementary Content). Famine also persisted into 941 CE at least in parts of the Benelux countries (Supplementary Content) and, according to later sources, Germany (Zusätze Röchell’s zu Frühern Chronisten 1856, p.187–8). Parts of France, Switzerland and the Benelux countries were afflicted still in 942 CE when Flodoard of Rheims wrote in his *Annales* of a ‘great food shortage through all Francia and Burgundia’ (Supplementary Content). Dearth and subsistence crises also visited the Baghdad area from 940 to 941 CE (Ibn al-jawzi 1992; Bar Hebraeus 2003) and the Maghreb (Skylitzes 2010).

As indicated, hardship accompanied the uncommonly cool temperatures experienced in eastern China as well. In mid-May 941 CE, there are reports of both widespread starvation and public relief efforts in Henan and Shandong provinces, which could suggest an uneven occurrence of harvest failure (regions without public granaries suffered more), and in winter 941/942 CE of starving soldiers (Supplementary Content). Extensive flooding reported in eastern China in the spring of 941 CE, possibly fuelled by heavy winter snowfall, and inundations in the autumn of the same year undoubtedly aggravated food shortages. Flooding recurred in the late spring of 942 CE, which may indicate that winter 941/942 CE was unusually long (Supplementary Content).

Although historical sources do not record pronounced aridity in Europe, the Old World Drought Atlas identifies exceptionally dry conditions north of the Alps in the summers of 942–944 CE (Cook et al. 2015). Severe spring–summer drought conditions are observed widely in eastern China in 942–943 CE as well. At the same time, there were locust infestations both in Central Europe in 941 CE (Zusätze Röchell’s zu Frühern Chronisten 1856, p.187) and in the Yellow River basin in 942–943 CE (Supplementary Content). In 943 CE, famine inflated foodstuff prices and families migrating for want of food are referenced alongside reports of tens of thousands of deaths in eastern China. Hundreds of thousands of deaths were reported the following year (Supplementary Content). The European evidence is terser and less speculative, but subsistence crises there likely contributed greatly to human mortality and to a vast cattle plague, exacerbating the agricultural consequences of summer cooling (Newfield 2015). It appears that multiple environmental anomalies, potentially synergistic, coalesced to challenge the subsistence strategies of contemporaries in many and disparate regions. In Europe, the Middle East and China, the conditions for famine and mortality mounted over a number of years after the Eldgjá eruption.

### *Vǫluspá* and the Eldgjá eruption

Returning to the eruption itself, we focus on one of Iceland’s most celebrated medieval poems, the apocalyptic *Vǫluspá* (‘the prophecy of the seeress’). It is included in the *Codex Regius* (Fig. 1c), written circa 1270 CE, but recapitulating much earlier poetry and Scandinavian and Germanic folklore (Orchard 2011). We suggest it contains literary references to the Eldgjá eruption and that these assume wider significance in the context of Iceland’s conversion to Christianity. The poem describes how the revered pagan god Odin raises a prophetess from the dead. She foretells the end of the pagan pantheon and the coming of a new (and singular) god in a series of portents, one being the rearing of a monstrous wolf that will swallow the Sun. The descriptions, found notably in stanzas 41 and 57, are strongly suggestive of an eruption of the magnitude, duration and likely impacts of the Eldgjá eruption:

**Table:** 

41: Fylliz fjǫrvi feigra manna, rýðr ragna sjǫt rauðum dreyra; svǫrt verða sólskin of sumur eftir, veðr ǫll válynd. Vituð ér enn—eða hvat?	[The wolf] is filled with the life-blood of doomed men, reddens the powers’ dwellings with ruddy gore; the sun-beams turn black the following summers, weather all woeful: do you know yet, or what?
57: Sól tér sortna, sígr fold í mar, hverfa af himni heiðar stjǫrnur. Geisar eimi ok aldrnara, leikr hár hiti við himin sjálfan.	The sun starts to turn black, land sinks into sea; the bright stars scatter from the sky. Steam spurts up with what nourishes life, flame flies high against heaven itself.

The poetic circumlocution ‘what nourishes life’ from stanza 57 is generally taken to refer to fire. The two stanzas taken together, describing an explosive and fiery event reaching up to the sky in which the air above first turns blood-red, while the land seems to recede, and then the Sun and other heavenly bodies are obscured by darkness over several years, suggest a close literary interpretation of what might have been observed at first hand when Eldgjá erupted (see also Supplementary Content). Referring to stanza 41, the Icelandic mediaevalist, Sigurður Nordal, who edited *Vǫluspá*, noted that ‘I do not think I have ever understood this description until I saw volcanic ash falling in a clear sky from the eruption of Katla in 1918. The sun was shining then, but it was dark, as was all its shine. Impressive as a total eclipse of the sun is (as popular beliefs and folktales witness), this was yet much more grim and terrible’ (Nordal 1923). Thorarinsson (1981) also recognised the volcanic allusions in the poem but suggested that these were references to an eruption of Katla in 1000 CE.

Further lines in the poem suggest volcanic manifestations. In stanza 38, ‘venom-drops flowed in through the roof-holes’, which may recall acid rain associated with volcanic plumes. This finds an echo in Pastor Jón Steingrímsson’s account of the Laki eruption: ‘more poison fell from the sky than words can describe: ash, volcanic hairs, … rain full of sulphur and saltpetre…’ (Steingrímsson and Kunz 1998). In stanza 52, there is a reference to the fire giant ‘Surt comes from the South with what damages branches’. In verse 60, the gods are said to come together at their home to commemorate these ‘mighty events’. The fact that the devastation of the mythical fire-giant Surt is said to ‘come from the South’ maps on to the geographical location of Eldgjá, which is even now designated as situated in Suðurland (‘the southern part of the country’).

If our attribution of these parts of *Vǫluspá* to manifestations and climatic consequences of the Eldgjá eruption is correct, our secure date for the eruption implies that *Vǫluspá* was composed after 939/940 CE.

#### The Christianisation of Iceland

In calling attention to experiences and memories of the Eldgjá eruption as signs that the old pagan ways were doomed, *Vǫluspá* suggests that the eruption acted as a catalyst for the profound cultural change brought about by conversion to Christianity. While this was a gradual process over the latter half of the tenth century, and there existed a Christian population of Celtic roots even since the settlement of Iceland, the official date for conversion (*kristnitaka*) is 999/1000 CE. This followed a heated national parliament at Þingvellir (where annual gatherings had been held since 930 CE, before the Eldgjá eruption). In *Kristni saga* (‘the story of the conversion’), written in the thirteenth century, one of the key events is the sudden arrival of a man reporting a volcanic eruption. He claims that lava is threatening the land of one of the chieftains, Þóroddr of Ölfus, who was evidently on the Christian side. When the pagans noted that this was a sign of the gods’ displeasure, another powerful chieftain, pointing to the dramatic landscape of Þingvellir itself, asks ‘What were the gods enraged by when the lava we are standing on here and now was burning?’ (Grønlie 2006). Later traditions evidently associated the conversion with volcanic events, just as *Vǫluspá* does, in that case likely the Eldgjá eruption itself.

## Conclusions and final remarks

Through the serendipity of the 775 CE solar particle event being recorded in a tree killed by the Millennium Eruption of Changbaishan volcano, and the high-resolution glaciochemistry of the NEEM-2011-S1 ice core, we have been able to constrain the onset of the Eldgjá eruption to circa spring 939 CE. The eruption persisted, likely episodically, at least until around autumn the following year. Aspects of the deposition of volcanic materials to the Greenland ice surface, as recorded in both the NEEM-2011-S1 and GISP2 ice cores, are puzzling and warrant further investigation. In particular, highly depth-resolved analysis of tephra and volatile species in various arctic ice cores, including sulphur isotope measurements, might reveal insights into both volcanic source conditions and atmospheric chemistry and transport of the volcanic plumes.

Abundant medieval sources attest to the spread of atmospheric haze associated with the eruption, and many contemporary sources are suggestive of climatic effects (severe winters in 939/940 and 940/941 CE) and societal consequences (abandonments, food shortage, animal mortalities) in Europe and China. In their similarity to some of the experiences following the eruption of Laki in 1783–1784, these findings reinforce our understanding of the long-range impacts of lava flood eruptions.

Since the traditional date for the Settlement of Iceland is 874 CE, and the initial migration is supposed to have been completed within 60 years, dating the Eldgjá eruption to 939–940 CE places it squarely within the experience of the first two or three generations of settlers: indeed, it is plausible that some of the first wave of migrants, brought over as children, witnessed the eruption.

Our linking of lines in the apocalyptic poem, *Vǫluspá*, to experiences of Eldgjá and its aftermath, and the accepted literary interpretation of the poem as a prophecy of the demise of the pagan gods, focuses attention on the eruption’s significance in the context of Iceland’s Christianisation in the latter decades of the tenth century. We should recall that the effects of the 1783–1784 Laki eruption and associated ‘haze famine’ were devastating for Iceland—more than a fifth of the population perished within a couple of years. The early colonists in the tenth century are unlikely to have fared better during the Eldgjá eruption, nor, given their Scandinavian and British Isles ancestry, would they have been as familiar with volcanism as Icelanders were by the eighteenth century (Hartman et al. 2017). Recalling, too, that the Laki eruption was seen by contemporary observers as retribution from God (e.g. Steingrímsson and Kunz 1998), and the association of volcanic ‘fires’ with the tortures of Hell, a role for the Eldgjá eruption in fuelling theological debate and proselytisation in the tenth century seems plausible.

## Electronic supplementary material

ESM 1 (DOCX 273 kb)
