# Supplementary material for: The Eldgjá eruption: timing, long-range impacts and influence on the Christianisation of Iceland
Source: Clim Change. 2018 Mar 19;147(3):369–81. doi: 10.1007/s10584-018-2171-9 (PMC6560931; doi:10.1007/s10584-018-2171-9)
Supplement: Supplementary file 1 — (DOCX 273 kb) [file 10584_2018_2171_MOESM1_ESM.docx]

Supplementary content

The Eldgjá eruption: timing, long-range impacts and influence on the Christianisation of Iceland

Clive Oppenheimer^a^*, Andy Orchard^b^, Markus Stoffel^c,d^, Timothy P. Newfield^e^, Sébastien Guillet^c^, Christophe Corona^f^, Michael Sigl^g^, Nicola Di Cosmo^h,i^ Ulf Büntgen^a,j,k^

1. Department of Geography, University of Cambridge, Cambridge, United Kingdom.
2. Faculty of English, University of Oxford, Oxford, United Kingdom.
3. Institute for Environmental Sciences, University of Geneva, Geneva, Switzerland.
4. Dendrolab.ch, Department of Earth Sciences, University of Geneva, Geneva, Switzerland.
5. Departments of History and Biology, Georgetown University, Washington, DC, USA.
6. Geolab UMR6042 CNRS, Université Blaise Pascal, Clermont-Ferrand, France.
7. Paul Scherrer Institut, Villigen, Switzerland.
8. Institute for Advanced Study, Princeton, NJ, USA.
9. Princeton University, Princeton, NJ, USA.
10. Swiss Federal Research Institute WSL, Birmensdorf, Switzerland.
11. Global Change Research Centre and Masaryk University, Brno, Czech Republic.

*Corresponding author; e-mail: [co200@cam.ac.uk](mailto:co200@cam.ac.uk); Tel.: +44 1223 333392; orcid.org/0000-0003-4506-7260

# Northern hemisphere summer temperature reconstructions around the 1783–4 Laki eruption

Figure S1. Comparison with Fig 3c-e – reconstructed summer temperatures around time of 1783–4 CE Laki eruption.

# 2. Medieval sources suggestive of atmospheric optical phenomena

## 2.1. The Chronicon Scotorum

The *Chronicon Scotorum* (also known as the “Chronicle of the Scoti or Scots”) only survives in a single paper manuscript (Trinity College Library, Dublin, 1292 formerly H. 1. 18) from circa 1640, which is ostensibly a faithful copy (transcribed by Dubhaltach Mac Fir Bhisigh, also known as Duald Mac Firbis, died 1671 CE) of a much earlier manuscript. It preserves a record of events from prehistory to 1135 CE, evidently compiled in a monastic milieu (Hennessy, 1886). For most of the tenth century, the text was kept at Clonmacnoise or Clonard in central Ireland (Evans, 2010: 89-90). Once the more fanciful aspects of the very patchy earliest entries are excluded, and the idiosyncrasies of reconciling the dual dating system are addressed (Walsh, 1941; McCarthy, 1998), the bare records for individual years from 442 to 1133 CE can be shown to be accurate in all cases where external validation is possible. This is particularly the case for astronomical observations such as eclipses and comets.

The individual entries in the *Chronicon Scotorum* are mostly concerned with events such as the deaths of prominent figures (especially bishops and kings); battles and killings (increasingly so as the Viking incursions intensified); disasters (notably plagues); and unusual natural phenomena. The last group is the smallest, and is also the one that seems most likely to have been based on first-hand observation. Both solar and lunar eclipses are routinely recorded, and a blood-red appearance of the moon is noted in the *Chronicon Scotorum* for the years 673, 692, and 807 CE, in each case following a litany of political events. An extraordinary aspect for the entry quoted in the main text for the year 939 CE is that it begins the account of the entire year. Given that portents in the sky, especially in a monastic context, were held to presage dire events on Earth, it is also notable that the entry lacks portentous connotations. Viking attacks are recorded both before and after, but no connection suggested. We are left with an eyewitness account of a remarkable atmospheric optical phenomenon that, in view of the strictness of monastic hours, appears to have been observed over at least 30 hours.

## 2.2. Annales Casinates

The Annales Casinates are a collection of fourteen 10^th^- and 11^th^-century notices from the Benedictine abbey of Monte Cassino, southeast of Rome, Italy. They were written by an anonymous chronicler and they span the period 915–1042 CE. The first notice is dated to 914 but is a mistake since it refers to the battle of Garigliano, fought in June 915 CE. There are three entries for the 930s CE. For 931 CE, we read of a newly renovated and rededicated altar and in 934 CE of the death of abbot Johannes (Annales Casinates, 1839). The entry for 938 CE is of a different sort: ‘Twelfth indiction, thirteenth day of the month of July, sixth festival, twenty-ninth moon, the sun was obscured from the third hour until almost the fifth hour. We looked at the sun, it did not have any strength, neither light nor heat. But we saw the sky and the colour [or appearance] of it changed, as though viscous. And others said that they saw the sun as though half’ (‘*ind. 12, 13. die stante mense Iulio, feria 6. Iuna 29. obscuratus est sol ab hora tertia usque pene ora 5. Aspiciebamus nos solem, non havebat ullam fortitudinem, nec ad splendorem, nec ad calorem; videbamus vero coelum, et mutatum erat color illius, tamquam libidus; et alii dixerunt, ut viderent solem tamquam dimidium’*) (1839, p. 172). The manuscript date is misleading. The twelfth indiction spanned September 938 CE to August 939 CE. Although several authors consider this a reference to a solar eclipse, Kostick and Ludlow (Sigl et al 2015, Supplementary Data) proposed the second half of the passage (from ‘We looked’) concerns an unrelated atmospheric phenomenon. Most plausibly, the Eldgjá eruption in spring 939 CE caused the Sun to appear weak and the sky viscous that summer in central Italy.

## 2.3. Widukind of Corvey

The *Res Gestae Saxonicae* were written by the Benedictine monk, Widukind of Corvey (*circa* 925 to after 973 CE) at the abbey of Corvey, east of Paderborn, Germany. The text was largely written in the mid-960s CE from earlier material. At one point, Widukind relates that many prodigies occurred before the death of the Ottonian King Henry I the Fowler (2 July 936 CE) ‘so that outside, the sky without a cloud [or ‘with a clear sky’], the light of the Sun was almost not visible, however inside it poured in through the windows of houses red as blood’ (‘*ita ut solis splendor forinsecus aere absque nubilo pene nullus appareret intrinsecus autem per fenestras domorum rubeus tamquam sanguis infunderetur*’; Widukind of Corvey, 1935, II.32, p. 93). Widukind sandwiches this observation between accounts of comets witnessed between 18 October and 1 November 941 CE and a cattle plague, which can be dated, using other sources, to 940–942 CE (see following).

# 3. Medieval European sources suggestive of climate change, dearth and mortality

940 CE *Hiemps valida et mortalitas animalium*. A strong winter [that of 939/40 CE] and a mortality of animals (Annales Colonienses, p. 98). This entry is dated in this thin and fragmentary work to 939 CE, a year early. The passage is succeeded in the text by a notice of the death of Rumold, Münster’s bishop, which is correctly dated to 941 CE. It is preceded by a notice of the 936 CE death of Henry I the Fowler, which is dated a year early to 935 CE. This imprecision, combined with the fact that other texts date the hard winter and animal die-off to 940 CE, suggests 940 CE is the correct date.

940 CE *Hiemps valida et mortalitas animalium facta*. A strong winter [that of 939/40 CE] and a mortality of animals occurred (Chronicum Suevicum Universale, p. 67).

940 CE *Annus durus et deficiens fructus*. A hard year and deficient produce (Annales Sangallenses, p. 78).

940 CE *Hyemps valida. Comete vise sunt. Mortalitas iumentorum*. A strong winter [that of 939/40 CE]. Comets were seen. A mortality of traction animals (Annales Capituli Cracoviensis, p. 15).

940 CE *Hiems saeva hoc anno facta et pestis animalium subsecuta*. The winter was violent in this year and a plague of animals followed (Hermann of Reichenau, p. 113).

940 CE *Necem ducum asperrima hiemps hiememque secutus est fames validissima*. The roughest winter [that of 939/40 CE] followed the death of the dukes and the strongest food shortage followed the winter (Widukind of Corvey, p. 89).

941 CE *Gaill i n-Inis Mochta iar lec ega gur oirgsiod hí*. Foreigners went into Inis-Mochta, over the ice, so that they plundered it (Hennessy, p. 203). Hennessy dates this passage of the Chronicon Scotorum to 940 CE, a year late. The church of Inis-Mochta, now Inishmot in Meath, Ireland was, in the Middle Ages, on an island in a lake. The winter in question is that of 940/41 CE.

941 CE *There was such Drouth and Ise over Ioghs & the waters of Ireland this yeare that the Danes went to Inis Moghty upon Ice & spoyled and ransacked the same* (Annals of Clonmacnoise, p. 152). This passage, clearly related to the above passage in the Chronicon Scotorum, is dated erroneously to 934 CE in the Annals of Clonmacnoise. The winter in question is that of 940/41 CE.

941 *Secc mor combtar suirissi locha & srotha*. Great frost, so that lakes and rivers were passable (Annals of Ulster, p. 461). Hennessy dates this passage to 940 CE, a year in arrears. The winter in question is that of 940/41 CE.

941 CE *Sed et in Italia octo continuis noctibus mirae magnitudinis cometa apparuit, nimiae proceritatis igneos ex sese radios fundens, subsecuturam non multo post famem portendens, quae magnitudine sui misere vastabat Italiam*. But also in Italy for eight continuous nights a comet of amazing greatness appeared, shedding from itself fiery rays of extreme length, portending the food shortage that would follow not long after, which with its greatness sadly devastated Italy (Liudprand of Cremona, V.2, p. 877). Luidprand does not date this passage. The Lombard historian references the comet and dearth after Ramiro II’s victory over Abd al-Rahman at Sinmancas (939 CE) and before Hugh of Italy’s appointment of Anscar of Spoleto, who died in 940 CE, as Duke of Spoleto (936 CE). The comet-dearth concurrence ties this passage to the others listed here, however, and allows us to date this food shortage to 941 CE.

941 CE *Signum mirabile apparuit in coelo et mortalitas boum fuit*. A marvelous sign appeared in the sky and there was a mortality of cattle (Annales Sangallenses, p. 78).

941 CE *Cometes apparuit. Et fames subsecuta*. Comets appeared. And a food shortage followed (Annales Leodienses, p. 16).

941 CE *Huius temporibus fuit maxima fames in universa terra. Et visus est cometa aperte mire magnitudinis et longitudinis*. In this time there was the greatest food shortage in all the land. And a comet of amazing size and length was seen clearly (Florenz von Wevelinkhoven, p. 12).

941 CE *Stella cometes apparuit et fames subsecuta est*. A comet appeared and a food shortage followed (Annales Lobienses, p. 234). This entry is erroneously dated to 943 CE in the text but the comet-dearth concurrence firmly dated to 941 CE in other texts demonstrates that the *Annales Lobienses* is a couple of years late here. This text also dates the death of King Henry I the Fowler to 938 CE (it was in 936 CE).

941 CE *Sed cometas inundatio nimia inundationemque boum pestilentia subsecuta est*. But an extensive flood followed the comets and a pestilence of cattle [followed] the flood (Widukind of Corvey, p. 93-94).

941 CE *Sidus simile cometae per XIIII noctes visum et immensa mortalitas boum secuta est*. A comet-like star was seen for 14 nights and a vast mortality of oxen followed (Adalbert of Magdeburg, p. 162). This passage is dated to 942 CE, a year early considering the firm comet-dearth date of 941 CE found in other passages listed here.

941 CE *Cometae 14 noctes apparent et mortalitas animalium facta*. Comets appeared for 14 nights and a mortality of animals occurred (Chronicon Suevicum Universale, p. 67). This passage is also dated to 942 CE in the text, a year early considering the firm comet-dearth date of 941 CE found in other passages listed here.

941 CE *Armenta moriebantur late*. Traction animals [cattle] died generally (Annales Iuvavenses, p. 743).

942 CE *Fames*. A food shortage (Annales Sancti Quintini Veromandensis, p. 507).

942 CE *Fames magna per totam Franciam et Burgundiam mortalitas quoque maxima boum grassata est in tantum ut valde pauca huiusmodi animalia in his remanserint terris*. There was a great food shortage through all Francia and Burgundia, also the greatest mortality of cattle spread so greatly that very few animals of this type remained in these lands (Flodoard of Rheims, p. 389).

942 CE *Fames valida fuit late*. There was a strong food shortage generally (*Annales Iuvavenses*, p. 743). This is a thin and discontinuous text. This food shortage is dated to 943 CE. Many events listed circa 940 CE are dated correctly (including King Henry’s 933 CE defeat of the Magyars (Hungarians), the 937 CE death of the Bavarian Duke Arnulf, and the 941 CE animal mortality) but some are not (for instance, Duke Berthold’s 943 CE defeat of the Magyars is dated to 942 CE, and the 947 CE death of this duke is dated to 948 CE). It appears the text is a year behind in the mid-940s CE. 942 CE is likely the correct date of this food shortage.

# 4. Medieval Chinese sources suggestive of climate change, dearth and mortality

A fresh survey of the Chinese sources has been conducted. Other surveys are presented in Fei and Zhou (2006, 2016).

939 CE二年 … 六月丁丑，雨雪。是夏，駐蹕頻蹕淀(*Liaoshi*, 4, p. 46). On 26 June, 939, it rained and snowed (in eastern Inner Mongolia). That summer, when clearing the passage for the imperial tour, people frequently swept water off the roads.

940 CE 十二月丁酉朔，百官不入閣，大雪故也。… 丁巳，帝謂宰臣曰：「大雪害民，五旬未止，京城祠廟，悉令祈禱，了無其驗 (*Jiu Wudaishi* 78, pp. 1033-34). On 12 January, 940, a hundred officials could not attend the *ruge* ceremony because of a heavy snowfall. On February 1, the emperor addressed the officials, saying: “The great snowfall has harmed the people; it has continued uninterruptedly for 50 days. We have ordered prayers and sacrifices in all temples of the capital, but they have been completed without results.”

941 CE (天福六年春正月) 乙丑，青州奏，海凍百餘里 (*Jiu Wudaishi* 79, p. 1045 ) (3 February, 941) from Qinzhou it was memorialized that the sea froze over a hundred li.

941 CE四月… 乙巳，齊、魯民饑，詔 兗、鄆、青三州發廩賑貸(*Jiu Wudaishi 79*, p. 1047). On 14 May, the people of Qi and Lu [Shandong and Henan] were starving. It was ordered that the three districts of Yan, Yun and Qing should provide relief from the public granary**.**

941 CE五月 … 庚午，涇州奏，雨雹，川水大溢，壞州郡鎮戍二十四城 (*Jiu Wudaishi 79*, p. 1047) On 8 June, Jingzhou memorialized that there was rain and hail, and the river’s water was greatly overflowing. Twenty-four city walls at the district, county, prefecture and garrison levels were broken**.**

941 CE 九月 … 辛酉，滑州河決，一溉東流，鄉村戶民攜老幼登丘冢，為水所隔，餓死者甚眾 (*Jiu Wudaishi 80*, p. 1053). On 27 September, the river in the Hua Prefecture [present day Henan province] broke the banks. The floodwaters flowed eastward, people from villages and hamlets carried the old and young to climb on hillocks and mounds, were cut off by the water, and masses of people died of hunger.

941 CE是冬大寒，潰兵飢凍及見殺無孑遺 (*Xin Wudaishi* 51, p. 585). That winter (941-942) was extremely cold. Scattered soldiers were starving and freezing and one could see those killed and no survivors left behind.

941 CE 九月…丁丑...河決中都，入于沓河。(*Xin Wudaishi* 8, pp. 85-86). On 13 October, the river flooded the central capital and entered it (divided) into many rivers

941 CE會同 四年 .. 冬十月辛丑，有司奏燕、薊大熟(*Liaoshi* 4, p. 50). In winter, on 6 November, it was reported to the throne that there were great storms in Yan and Ji [the region north of the Gulf of Bohai].

941 CE天福六年… 十一月, 甲申 … 唐主欲遂居江都，以水凍，漕運不給，乃還*(Zizhi Tongjian* 282, p. 9218). On 24 December, 941, the Lord of Tang wished to move his residence to Jiangdu [in today’s Jiangsu province], but because the [Grand Canal] water had frozen, water transport could not be provided, and he had to turn back.

942 CE 是春，鄴都、鳳翔、兗、陝、汝、恆、陳等州旱，鄆、曹、澶、博、相、洺諸州蝗。(*Jiu Wudaishi* 80, p. 1059). That spring, Ye City and the prefectures of Fengxian, Yan, Shan, Ru, Heng, Chen and others had droughts; the prefectures of Yun, Cao, Chang, Bo, Xiang and Ming all had locusts.

942 CE 是月[五月] 州郡五奏大水，十八奏旱 蝗. (*Jiu Wudaishi* 80, p. 1061) That month [17 June ~ 15 July, 942] five prefectures reported a flood and eighteen reported drought and locusts.

942 CE從幸鄴都，是歲遇旱，高祖遣祈雨於白龍潭，有白龍見於潭心，是夜澍雨尺餘，人皆異之 (*Jiu Wudaishi* 81, p. 1068). [Emperor Shaodi] conducted an inspection of Yedu. That year had met with drought. So Gaozu (previous emperor) had sent people to pray for rain at the White Dragon Lake. White dragons were seen at the center of the lake, and that night over a foot (30 cm.) of rain fell. Everyone found this extraordinary.

942 CE承福以其兵從出帝禦虜。是歲大熱，吐渾多疾死，乃遣承福歸太原，居之嵐、石之間。(*Xin Wudaishi* 74, pp. 910-011). [Bai] Chengfu [leader of Tuyuhun] with his troops joined the Emperor in an expedition against the enemy. That year [942] it was extremely hot and many Tuyuhun fell ill and died. Therefore [the emperor] sent Chengfu back to Taiyuan, to settle them between mountains and rocks [for relief from the heat].

942 CE十一月…戊戌，詔宰臣等分詣寺廟祈雪 (*Jiu Wudaishi* 81: 1073). On 28 December 942 an imperial decree ordered that prefects, ministers and other officials separately visit various temples and shrines and pray for snow.

943 CE河中逃戶凡七千七百五十九。是時天下饑，穀價翔踴，人多餓殍 (*Jiu Wudaishi* 81, p. 1075). In the second month [9 March ~ 7 April 943] altogether 5,759 families fled Hezhong [in southern Shanxi]. At that time, there was famine throughout the empire, the price of grain soared sky-high, and many people starved.

943 CE [夏四月] … 是月，河南、河北、關西諸州旱蝗，分命使臣捕之 *Jiu Wudaishi* 81, p. 1076). In the fourth month, all the districts of Henan, Hebei, and Guanxi had drought and locusts. It was separately ordered to the various officials to catch them.

943 CE 八月…辛亥，分命朝臣一十三人分檢諸州旱苗。涇、青、磁、鄴都共奏逃戶凡五千八百九十。(*Jiu Wudaishi* 82, p. 1081). On 7 September 943, it was separately commanded that thirteen court officials separately examine all the prefectures for dried out sprouts. The cities of Jing, Qing, Ci and Ye collectively memorialized that 5890 families had fled.

943 CE 是冬大飢，河南諸州餓死者二萬六千餘口(*Jiu Wudaishi* 82, p. 1084) That winter there was a great famine, over 26000 people in every district of Henan starved to death.

943 CE是歲，春夏旱，秋冬水，蝗大起，東自海壖，西距隴坻 。南踰江、淮北抵幽薊(*Zizhi Tongjian* 283, p. 2257). That year, there were droughts in spring and summer, and in autumn and winter there were floods and locusts, in the east from the sea littoral, in the west up to the slopes of the Long mountain, in the south down to the Yangtze and Huai rivers, and in the north extending to faraway Ji [near Beijing].

944 CE是歲，天下餓死者數十萬人，詔逐處長吏瘞之(*Jiu Wudaishi* 82, p. 1085). That year, several hundreds of thousands of people starved to death in the empire. An imperial decree ordered that chief and functionary in each and every locality bury them.

944 CE 夏四月…丁巳 … 同、華奏，人民相食。丙寅，隴州奏，餓死者五萬六千(*Jiu Wudaishi* 82, pp. 1089-1090). On 10 May 944, Tong and Hua prefectures memorialized that people were eating each other. On 19 May 944, the Long prefecture memorialized that 56,000 people had starved to death

944 CE是月，澤潞上言，餓死者凡五千餘人(*Jiu Wudaishi* 82, p. 1090). In the fifth month, the Ze and Lu prefectures (in Shanxi) reported to the throne that over 5000 people had starved to death.

945 CE開運二年 … 二月 … 丙戌，幸鐵丘閱馬 … 是日大雪(*Jiu Wudaishi* 83, p. 1101). On 4 April 945, [the Emperor] went to Tiaqiu to insect the horses. […] That day there was a heavy snowfall.

945 CE 是時，天下旱蝗，晉人苦兵(*Xin Wudaishi*, 72, p. 895). At that time [945] the empire had droughts and locusts. The people of Jin suffered from the armies.

# 5. A note on Stothers (1998) and his date of 934 CE for the eruption

The date for the Eldgjá eruption that has been most widely cited in the literature is 934 CE, and follows the work. It is therefore worth examining his arguments. He cites the passage from Widukind of Corvey discussed here in the main text and 2.3 of the supplementary content. He links the passage not only with the Eldgjá eruption but with his preferred dating of 934 CE, as well as with the subsequent death of the Ottonian King Henry I the Fowler. This reading is problematic. An allegedly corroborating entry cited by Stothers **(1998).** comes from the *Annals of Clonmacnoise*, a seventeenth-century English translation of a lost Irish chronicle otherwise known as *Mageoghagan’s Book* (Hennessy, 1896, p. 151; Grabowski and Dumville, 1984). The passage ‘*The sunn for one day appeared like blood untill noone the next day*’ is dated to 933 CE in this work.

Stothers notes that: ‘chronology in the *Annals of Clonmacnoise* is not always completely accurate’. Not so for *Chronicon Scotorum*, which shares several entries with the *Annals of Clonmacnoise*, the alignment of which allows for the erroneous dating given for the *Annals of Clonmacnoise* entry to be corrected to 939 CE. In any case, both texts belong to the Clonmacnoise group of Irish annals and these passages are clearly interdependent; there is only one Irish observation of volcanic haze. Additionally, the exceptional winter cold alluded to in the *Annals of Clonmacnoise* at 934 CE, and noted by Stothers, is correctly dated, following the *Chronicon Scotorum*, to winter 940/941 CE (see preceding section). Finally, we note that the lack of cooling (based on analysis of both tree-ring records and documentary sources) in 934 or 935 CE provides further evidence against the eruption having taken place in 934 CE.

# References

Adalbert of Magdeburg, 1890, *Chronicon*, ed. F. Kurze *Monumenta Germaniae Historica SrG V* (Hanover): 154-179.

Annales Capituli Cracoviensis, 1866, ed. G. Pertz Monumenta Germaniae Historica SrG XI (Hanover): 9-43.

Annales Casinates (1839), ed. G. Pertz Monumenta Germaniae Historica SS III (Hanover): 171-172.

Annales Colonienses, 1826, ed. G. Pertz Monumenta Germaniae Historica SS I (Hanover): 97-99.

Annales Iuvavenses, 1934, ed. H. Bresslau Monumenta Germaniae Historica SS XXX.2 (Leipzig): 722-744.

Annales Leodienses, 1841, ed. G. Pertz Monumenta Germaniae Historica SS IV (Hanover): 9-20.

Annales Lobienses, 1881, ed. G. Waitz Monumenta Germaniae Historica SS XIII (Hanover): 224-235.

Annales Sancti Quintini Veromandensis, 1859, ed. L. Bethmann Monumenta Germaniae Historica SS XVI (Hanover): 507-508.

Annales Sangallenses Maiores Pars Altera, 1826, ed. G. Pertz Monumenta Germaniae Historica SS I (Hanover): 78-85.

*Annals of Clonmacnoise*, 1896, ed. Murphy D.S.J. (ed.), 1896. Dublin, Printed at the University Press for the Royal Society of Antiquaries of Ireland, 393p.

*Annals of Ulster I*, 1887, trans. W. Hennessy (Dublin).

Chronicon Suevicum Universale, 1881, ed. H. Bresslau Monumenta Germaniae Historica SS XIII (Hanover): 61-72.

Evans, N., 2010, The Present and the Past in Medieval Irish Chronicles (Martlesham).

Fei J, J Zhou (2006) The possible climatic impact in China of Iceland’s Eldgja eruption inferred from historical sources. Clim Change **76**:443-457.

Fei J, Zhou J (2016) The drought and locust plague of 942–944 AD in the Yellow River Basin, China. Quat Int 394:115-122.

Flodoard, *Annales*, 1839, ed. G. Pertz *Monumenta Germaniae Historica SS III* (Hanover), p. 363-407.

Florenz von Wevelinkhoven, 1851, Chronik der Bischöfe von Münster, ed. J. Ficker Die Geschichtsquellen des Bisthums Münster I: Die Münsterischen Chroniken des Mittelalters (Munster): 1-91.

Grabowski, D. N. Dumville. Chronicles and Annals of Medieval Ireland and Wales: the Clonmacnoise Group of Texts. Boydell (1984).

Hennessy, W.M., 1866, (ed. and transl.) Chronicon Scotorum. A Chronicle of Irish Affairs from the earliest times to AD 1135 with a supplement containing the events from 1141 to 1150. Rerum Britannicarum Scriptores, vol. 46, London, Longmans, Green, Reader and Dyer.

Hermann of Reichenau, 1844, *Chronicon*, ed. G. Pertz *Monumenta Germaniae Historica SS V* (Hanover): 67-133.

*Jiu Wudaishi*, 1976, comp. Xu Juzheng et al (Beijing).

*Liaoshi*, 1974, comp. Tuotuo et al (Beijing).

Liudprand of Cremona, 1853, *Antapodosis*, ed. J.-P. Migne *Patrologicae Latina CXXXVI* (Paris): 787-898.

McCarthy, D. P., 1998, The Chronology of the Irish Annals’. *Procs. Roy. Irish Acad.* **98C**, 203–255.

Stothers, RB, 1998, Far reach of the tenth-century Eldgjá eruption, Iceland. *Clim. Change* **39**, 715–26.

Walsh, P., 1941, The Dating of the Irish Annals. *Irish Hist. Stud.* **2**, 355–375.

Widukind of Corvey (1935), *Res Gestae Saxonicae*, eds. P. Hirsch and H.-E. Lohmann *Monumenta Germaniae Historica SrG LX* (Hanover). http://www.hs-augsburg.de/~harsch/Chronologia/Lspost10/Widukind/wid_sa2t.html

*Xin Wudaishi*, 1977, comp. Ouyang Xiu, Ann. Xu Wudang (Beijing).

*Zizhi Tongjian*, 1956, comp. Sima Guang et al, Ann. Hu Sanxing (Beijing).
